# New Bioengineering Breakthroughs and Enabling Tools in Regenerative Medicine

**DOI:** 10.1007/s40778-017-0081-9

**Published:** 2017-05-04

**Authors:** Alvaro Mata, Helena S. Azevedo, Lorenzo Botto, Nuria Gavara, Lei Su

**Affiliations:** School of Engineering and Materials Science, Institute of Bioengineering, Queen Mary University of London, London, E1 4NS UK

**Keywords:** Bioengineering, Regenerative medicine, Biomaterials, Mechanobiology, Modeling, Imaging

## Abstract

**Purpose of Review:**

In this review, we provide a general overview of recent bioengineering breakthroughs and enabling tools that are transforming the field of regenerative medicine (RM). We focus on five key areas that are evolving and increasingly interacting including mechanobiology, biomaterials and scaffolds, intracellular delivery strategies, imaging techniques, and computational and mathematical modeling.

**Recent Findings:**

Mechanobiology plays an increasingly important role in tissue regeneration and design of therapies. This knowledge is aiding the design of more precise and effective biomaterials and scaffolds. Likewise, this enhanced precision is enabling ways to communicate with and stimulate cells down to their genome. Novel imaging technologies are permitting visualization and monitoring of all these events with increasing resolution from the research stages up to the clinic. Finally, algorithmic mining of data and soft matter physics and engineering are creating growing opportunities to predict biological scenarios, device performance, and therapeutic outcomes.

**Summary:**

We have found that the development of these areas is not only leading to revolutionary technological advances but also enabling a conceptual leap focused on targeting regenerative strategies in a holistic manner. This approach is bringing us ever more closer to the reality of personalized and precise RM.

## Introduction

**Fig. 1:**
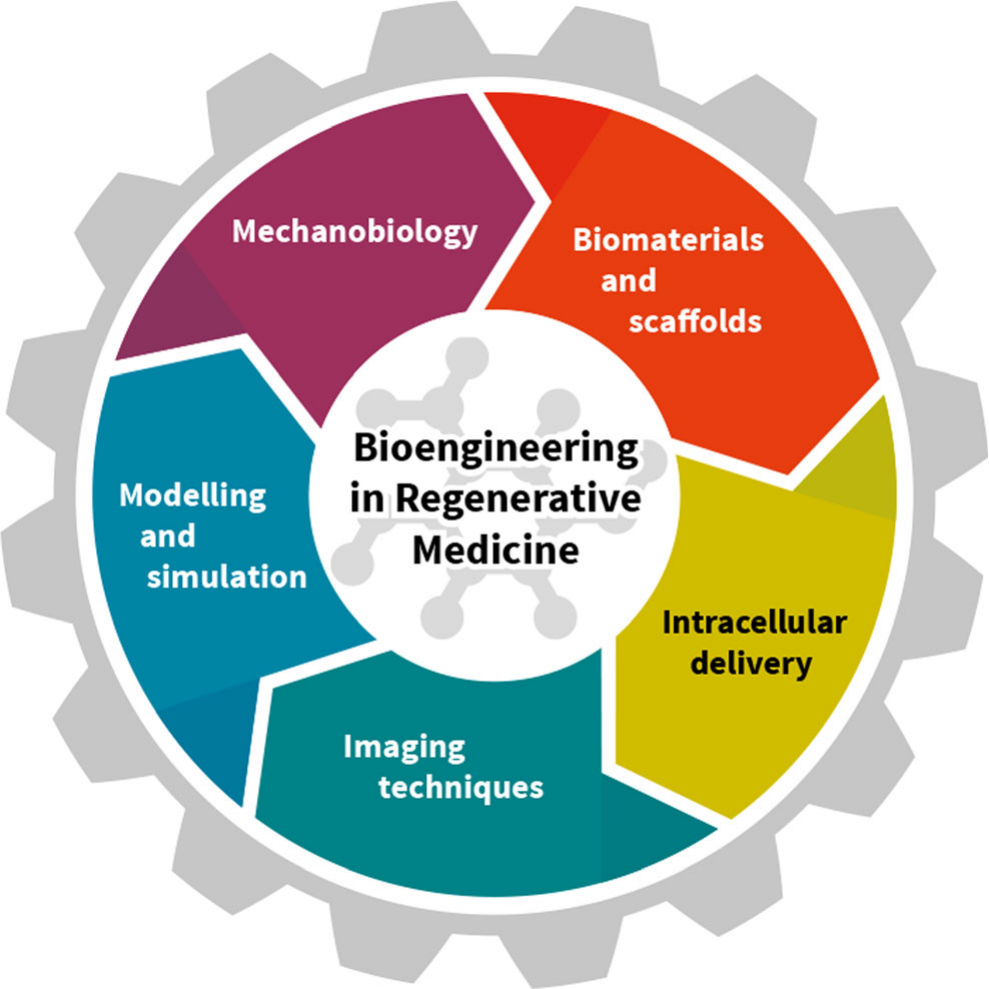
Summary of the focus of this review highlighting five key areas that are coming together to transform the field of RM

The increasing integration of traditional scientific disciplines such as materials science, chemistry, and biology and the emergence of research fields like synthetic biology, supramolecular chemistry, or mechanobiology continue to expand the field of bioengineering. Today, the field of bioengineering is a testament to the possibilities of interdisciplinary research. Regenerative medicine (RM) is a particularly interesting target for the development and application of novel bioengineering solutions. The inherent biological and molecular complexity, multi-scale organizations, and spatio-temporal features of regenerative processes can be tackled through an ensemble of technological angles. For example, most regenerative challenges can now be tackled through a holistic understanding of biological events, molecular design, selective monitoring or sensing, and the capacity to numerically simulate events to predict or optimize performance. This cooperative strategy is resulting in ever more integrated therapeutic approaches that are redefining the traditional view of implants, devices, drugs, or biomaterials. In this review, we attempt to provide a general overview of work being conducted in recent years in five key complementary areas of bioengineering including: mechanobiology, biomaterials and scaffolds, intracellular delivery, sensing and imaging, and computational and mathematical modeling (Fig. 1).

### Mechanobiology in Regenerative Medicine

In the body, cells encounter a dynamic environment. To respond to chemical and/or physical stimuli, cells reorganize their cytoskeleton and alter their function. The current paradigm states that cells have the ability to constantly probe their environment. They do so by exerting minute forces to sense the physical properties of their matrix. This process, known as mechanotransduction, takes place at the sites of cell adhesion and leads to the activation of signaling cascades to control cell function. Similarly, the lack of appropriate physical cues leads to altered cellular states, as observed when isolated cells are cultured in vitro in plastic dishes. In these conditions, several cell types dedifferentiate, and stem cells lose their self-renew and progressively enter senescence.

Matrix stiffness as relevant physical stimulus to control stem cell differentiation was first highlighted by Engler et al. [1]. Long-term culture of stem cells on matrices with stiffness similar to bone would upregulate genes and express protein characteristic of osteogenic differentiation. Similarly, matrices with stiffness of muscle or brain would result in myogenesis and neurogenesis, respectively. Such cell fate decisions require the modulation of myosin-II contractility. A plethora of studies later explored the use of other materials to generate matrices with tunable stiffness, including extracellular matrix (ECM) protein-based gels, polysaccharide-based alginate gels, and non-natural polymeric gels, such as polyacrylamide. Since the later are chemically inert, cell adhesion is enabled by coating matrices with ECM proteins. The validity of these results was questioned, due to the fact that the modulation of gel stiffness is obtained by changing the density of crosslinkers, which in turn alters surface porosity, geometry, and ligand-binding capabilities [2]. Nevertheless, in a rebuttal study, Engler and co-workers produced two families of polyacrylamide gels of constant stiffness (bone-like and fat-like stiffness), but with varying porosity or ligand-substrate tethering. Since differentiation was not affected by changes in porosity or tethering, the authors concluded that stiffness was the only cue directing cell fate [3]. An alternative and elegant solution was proposed by Fu et al. using micropost arrays of constant material composition but featuring different micropost heights [4]. By doing so, the authors decoupled substrate stiffness from surface properties, and observed similar differentiation patterns to those found using synthetic gels. Studies culturing cells inside 3D hydrogels have confirmed that matrix mechanics does also control cell fate in a cellular environment more similar to tissue. Huebsch et al. first reproduced in 3D alginate gels the results previously found in 2D polyacrylamide gels [5]. Again, when cultured within matrices whose stiffness mimicked bone or adipose tissue, stem cells secreted proteins typically associated with osteogenesis or adipogenesis, respectively. Nevertheless, recent findings have suggested that in 3D conditions, cell fate does not depend on matrix stiffness but rather on the cell’s ability to degrade its surrounding matrix, to then generate traction forces onto it [6].

Within tissue, cells are normally subjected to a variety of dynamic mechanical forces, such as fluid shear stress, tension, and (hydrostatic) compression. While a large number of studies have examined the role of mechanical forces on stem cell differentiation, it is difficult to draw general conclusions due to the multifactorial nature of mechanical stimuli. Mechanical loading regimes are defined by the type of load (strain, compression, shear), the magnitude, dimensionality (uniaxial or biaxial), loading frequency, and duration of the overall regime. As a result, no systematic study exists but rather a myriad of studies assessing the effect of a particular loading regime. To summarize briefly the main findings, cyclic strain induces osteogenesis [7, 8] or chondrogenesis [8], fluid shear stress favors osteogenesis [9], and vasculogenesis [9], while hydrostatic pressure or uniaxial compression favors chondrogenesis [10, 11].

Modulation of cell spread area and shape using micro-patterning is also considered within the repertoire of methods to control stem cell fate by physical means. Smaller cell spread areas or round patterns are associated with adipogenesis [12] or maintenance of stemness [13]. Larger cell spread areas [14] or cell shapes with concave edges [12, 15] that promote actin cable formation promote osteogenesis, with differentiation being associated with changes in cytoskeletal protein assembly and signaling via RhoA and Rock [14, 16]. Along these lines, disruption of actomyosin stress fibers by chemical means also leads to adipogenic or neurogenic differentiation [17–19]. Together, these results highlight the role of cytoskeletal tension as a key regulator of stem cell fate.

The mechanical connection between the cell and its environment is not restricted to the physical link between ECM, focal adhesions, and cytoskeleton. Rather, it extends deeper into the cell via a group of proteins, the LINC complex, that mechanically connect the cytoskeleton to the nucleus. It has been suggested that this physical link may serve as an efficient and fast relay of mechanical information directly to the nucleus via an “action at a distance” mechanism. Nevertheless, little is still known about the actual players of the mechanotransduction event and the proteins involved in converting the physical signal into a chemical signal. Candidate proteins include the YAP/TAZ pathway, which has been suggested as a hub for the conversion of mechanical information into chemical information. YAP/TAZ is broadly implicated in cell fate decisions, and studies using mechanical stimuli as diverse as modulation of cell spread area [20], matrix stiffness [21] or mechanical loading [22] have all identified YAP/TAZ as key player. Increased cytoskeletal tension is also associated with increases in laminA expression and its phosphorylation, resulting in translocation of retinoic acid receptor γ (RARG) to the nucleus [21] and the activation of the retinoic acid signaling pathway. Finally, increased actin polymerization levels (or specifically, the decrease in G-actin levels) have also been shown to induce differentiation by upregulating the activity of MAL and its binding to serum response factor (SRF) to activate its transcription [13].

A plethora of studies report similar results on stem cells from other origins, such as hematopoietic [23], adipose [24], epidermal [13], embryonic [25], and neural [26]. Recent studies have further assessed the fate and therapeutic capabilities of mechanically conditioned cells when used as a xenograft transplantation model [27]. Still, it remains to be understood whether mechanical conditioning is most efficiently performed ex vivo or in situ, and how far along the differentiation process is it best to transplant the cells into its target location.

There are still other avenues to explore concerning the physical properties of the cellular environment. Synthetic matrices are treated as purely elastic materials than can be fully described with a single mechanical parameter, Young’s modulus. Recent studies have explored the role of viscoelasticity as a cue for differentiation [28]. Similarly, other “advanced” mechanical behaviors such as non-linear elasticity (the fact that a material may feel stiffer when pulled harder) or mechanical anisotropy are prevalent in native tissue and should be considered as plausible mechanical cues in situ. Secondly, mechanical cues have been mostly designed as uniform, and even when mechanical loading is applied, a single loading regime is used throughout the experiment. Future studies should consider the possibility to dynamically tune the physical stimuli presented to cells as they progress down their differentiation route.

Current methods are still reliant on the ex vivo expansion in plastic vessels of the isolated stem cell population. Nevertheless, evidence suggests the concept of ‘mechanical memory’, that is, the fact that stem cells grown for long periods of time in a particular matrix stiffness (either soft or stiff) undergo commitment and cannot be later re-directed to a different lineage [1]. A similar mechanical memory has been observed when stem cells are stimulated with mechanical loading regimes [29]. This phenomenon should force us to re-examine all stages of stem cell-based therapies, from isolation, to expansion, pre-transplantation, and delivery, questioning the suitability of the mechanical environment experienced by stem cells in each of these steps.

### Biomaterials and Scaffolds for Regenerative Medicine

The increased capacity to design at the nanoscale is enabling the recreation of biological materials and opening the possibility to guide biological processes. This opportunity is especially attractive in RM and is being approached both from the bottom-up building with molecules [30] and from the top-down using advanced fabrication techniques [31]. Today’s biomaterials can be programmable, information-rich, reversible, molecularly designed or tissue-derived, bioactive, biomimetic, and/or capable of exhibiting multiple functions. In this section, we provide a taste of pioneering work with special focus on biomaterials with enhanced precision and potential functionality for RM applications.

#### Bioactive Biomaterials

**Fig. 2:**
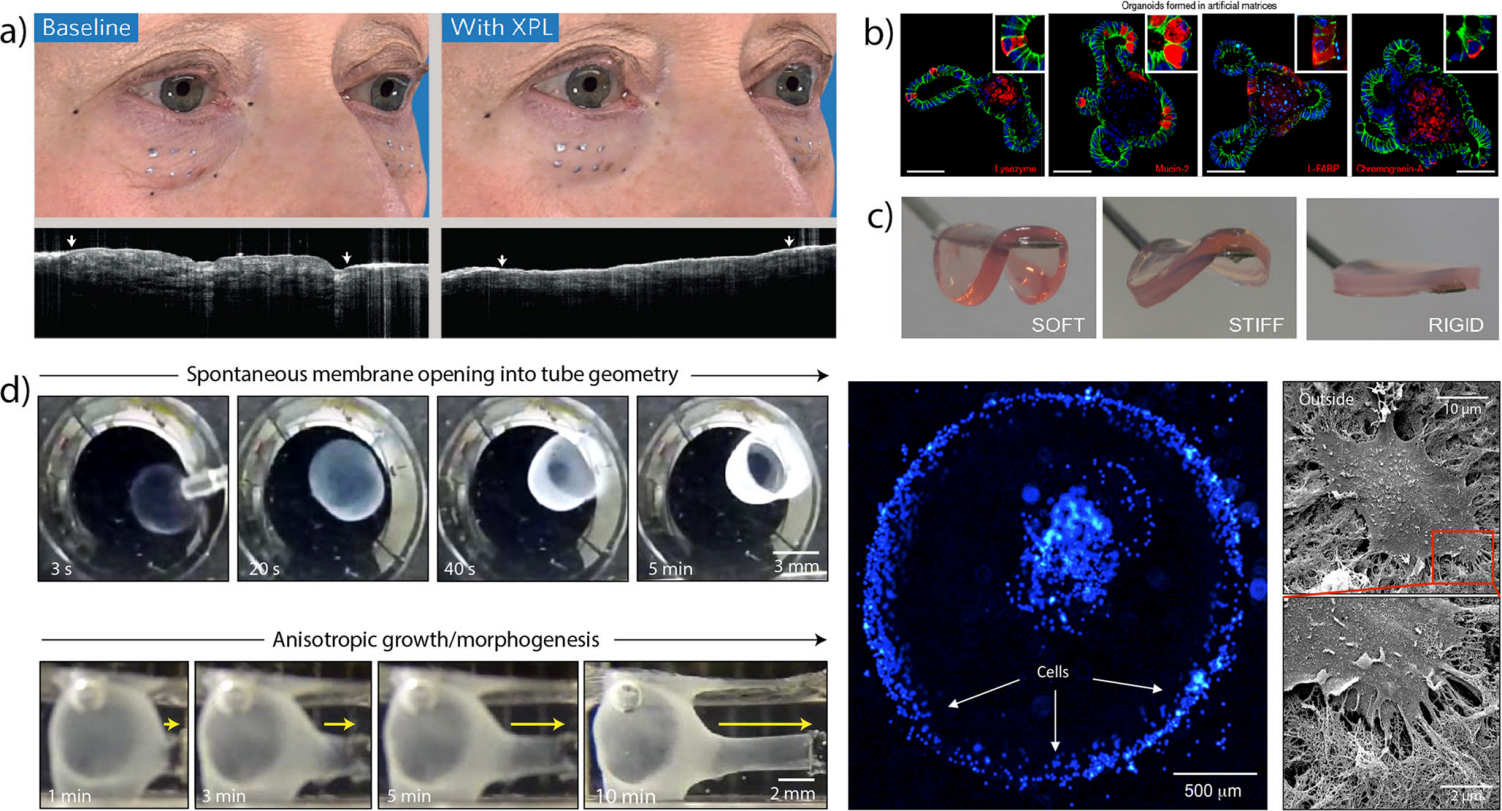
**a** Comparative image of the polysiloxane membrane serving as a “second skin” (XPL) capable of restoring natural function. (Reprinted by permission from Macmillan Publishers Ltd.: Yu B, et al. Nat Mater. 2016;15(8):911–8) [32]. **b** Organoid structures generated using a modular synthetic hydrogel with tuneable matrix elasticity and signaling properties. (Reprinted by permission from Macmillan Publishers Ltd.: Gjorevski, N, S et al. Nature. 2016;539(7630):560–4) [33]. **c** Hydrogels exhibiting tuneable stiffness based on self-assembling Fmoc-peptides. (From: Alakpa Enateri V, et al. Chem.1(2):298–319, with permission from Elsevier) [34]. **d** Dynamic self-assembly system to grow tubular vascular-like scaffolds (*yellow arrows* indicate the movement of a new anastomotic branch grown out of a main tubular structure, *red box* indicates a zoom view of mADSCs growing outside of the tubular structure after 7 days of incubation). (Reprinted by permission from Macmillan Publishers Ltd.: Inostroza-Brito KE, et al. Nat Chem. 2015;7(11):897–904) [35]

A major goal in RM is the capacity to stimulate biological responses with temporal and spatial control while exhibiting functional physical properties. With this in mind, Yu et al. developed a polysiloxane membrane that acts as a “second skin” and restores skin function thanks to its bulk elasticity, contractility, adhesion, and breathability (Fig. 2a) [32]. Material coatings can also provide such tissue-compatibility. For example, thin flexible perfluorocarbon layers have been developed to prevent thrombosis and formation of bacterial layers [36]. Functionality may also be enhanced not only by the properties of individual materials, but on their synergistic effect. A strong and tough hydrogel, a major biomaterial challenge, has been developed using an interpenetrating polymer network that interacts at the molecular scale to combine stiffness and brittleness with softness and elasticity [37]. Another example integrates modified tropoelastin and graphene oxide to create a hydrogel with both enhanced mechanical properties and conductivity with potential use in muscle regeneration applications [38]. Designing at the molecular scale, using for example recombinant technologies, facilitates the integration of mechanical properties and biomolecular signaling. For example, Tejeda-Montes et al., developed an elastin-like polymer membrane with outstanding mechanical properties [39] and the capacity to enhance osteoblastic phenotype and mineralization in vitro [40] as well as bone regeneration in vivo [41]. Molecular design enables a wide variety of biomolecular signaling relevant to tissue regeneration including for example regulation of the immune system [42], presentation of bioactive peptides [43], growth factor mimetics [44], or tuneable degradation [45].

#### Biomimetic Biomaterials

Tissue regeneration is characterized by an orchestrated set of dynamic processes where a wide variety of cells and structural and signaling molecules interact in a 3D space. In this scenario, recreation of the 3D space is essential and a number of excellent reviews address this goal [46, 47]. Despite tremendous work in this area, poorly defined animal-derived matrixes have been the material of choice of many when trying to recreate in vivo scenarios. It is essential to recreate the complexity of such environments but with reproducible and controllable tools. To this end, Lutolf and colleagues have pioneered modular synthetic hydrogel systems with tuneable matrix elasticity and signaling properties to study the modulation of cells (Fig. 2b) [33]. The past decade has seen a particular emphasis on the understanding of matrix stiffness to direct cell behavior, an exciting parameter to engineer therapies for RM. Recent studies have demonstrated the importance of stress-stiffening [48], stress relaxation [49], and 3D spatial confinement [50] of gels in guiding cell phenotype.

#### Self-Assembling Biomaterials

The last two decades have seen a growing interest in the development of supramolecular materials based on self-assembly due to the possibility to fabricate biomaterials that are modular and tuneable and can be systematically modified to enable properties such as responsiveness, bioactivity, and multifunctionality [51••]. Self-assembling systems based on peptides have been particularly popular and a large variety of excellent reviews have been published on this topic [52, 53]. The main advantage of these systems is the possibility to use bioinspired molecules, selectively interact with biological ones, and easily engineer systematic modifications to create materials that stimulate biological processes, recreate complex bioactive molecules, or tune environmental conditions. For example, using Fmoc-peptides, Alakpa et al., developed a simple self-assembling system with tuneable stiffness capable of directing cell behavior on demand (Fig. 2c) [34]. Others have used self-assembling nanofibers to selectively recruit and deliver growth factors (GFs) for bone regeneration [54], cells to treat ischemic cardiovascular diseases [55], or even mimic complex molecules such as growth factors [44] or glycosaminoglycans [56].

Future self-assembling systems are expected to provide a technological and functional leap that will take us beyond precise nanostructures and into dynamic materials exhibiting remarkable properties such as self-healing or the capacity to grow and adapt. These biomaterials will emerge from combinatorial approaches capable of optimizing molecular interactions through a natural selection of peptides [57] or proteins [58] and utilize dynamic intermolecular processes. For example, Inostroza-Brito et al., have introduced a peptide-protein supramolecular system capable of accessing non-equilibrium for substantial periods of time and enabling growth and morphogenesis into vascular-like tubular structures without the use of molds or templates (Fig. 2d) [35].

#### Biofabrication

The functionality of these materials for RM will also depend on the way they are processed. In additive manufacturing, for example, a large variety of bioinks are being developed with the goal of enabling bioactivity and biocompatibility while fulfilling critical processing requirements [59]. Novel biofabrication techniques are also enabling the development of complex hydrogel materials [60]. For example, photopatterning techniques can be used to generate chemically anisotropic regions containing patterns of peptides [61] or proteins [62]. Another versatile approach includes direct-writing fabrication, which has been used to pattern cellulose fibrils that give rise to dynamically reconfigurable hydrogels [63] or to print a hydrogel within a self-healing hydrogel to create anisotropic environments [64]. The capacity to create patterns within 3D soft matter is enabling targeting of a major challenge in RM, namely the generation of vascularized scaffolds. Examples include the use of multiphoton micromachining to create centimeter-deep vascularizing patterns [65] or bioprinting of multiple inks including cells, polymers, and hydrogels to create vascularized tissue-like structures [66]. A major functional breakthrough was recently reported by Kang et al., demonstrating the possibility to print various polymer and hydrogel-based inks with an integrated tissue-organ printer (ITOP) to create living human-size calvarial bone, cartilage, and skeletal muscle [67].

All these advances pave the way for an exciting future in biomaterials design. However, it is important to keep in mind that, while sophisticated materials continue to emerge, most current regenerative therapies continue to rely heavily on traditional materials. The necessary leap to transform these and other high-level technologies into functional therapies will require cohesive strategies that can stimulate both creativity and innovation and facilitate academic and industrial collaboration.

### Intracellular Delivery Strategies for Regenerative Medicine

Delivery strategies in RM are based on the controlled administration of molecular regulators—protein morphogens (e.g., GFs), nucleic acids (e.g., cDNA, mRNA, miRNA) or small molecules (e.g., retinoic acid, dexamethasone)—to specific cells or tissues to modulate cell fate, or improve the niche properties, and ultimately promote tissue regeneration. While some molecules exert their action extracellularly (e.g., GFs) by interacting with cell-surface receptors, nucleic acid therapeutics require internalization to access intracellular targets. Physical methods have been used to promote intracellular delivery of cargos, including microinjection, opto- or electroporation and cavitation [68]. However, these methods are quite invasive as they cause membrane disruption, being typically used in in vitro and ex vivo strategies. Clinically, less invasive and safer approaches are necessary. Carrier-based delivery systems are typically internalized by endocytosis and their physicochemical properties (size, shape, charge, ligand density) can be engineered to enhance the delivery of anabolic and catabolic factors into cells [69]. In this way, genes can be turned on or off to promote expression or inhibition of a certain protein involved in tissue regeneration. In this section, we highlight the use of bioengineering approaches to design multifunctional nanocarriers, through multivalency [70], targeting and cell-penetrating ability [71], stimuli-responsiveness [72, 73], and their application to promote selective intracellular delivery for optimized therapeutic outcomes.

#### Reprogramming Cells In Vitro

Cell reprogramming involves the delivery of specific transcription factors to activate endogenous genes in a cell resulting in its conversion into another cell type. A landmark in cell reprograming was reported by Yamanaka et al. who were able to convert mouse embryonic or adult fibroblasts into induced pluripotent stem cells (iPSCs) by delivering four transcription factors (Oct3/4, Sox2, Klf4, c-Myc) [74]. The transduced cells showed characteristics of pluripotency, indicating the possibility to generate PSCs from somatic cells for applications in RM. Since viruses have specific mechanisms to release genetic material inside cells, the transcription factors were delivered through retrovirus. Since the pioneering work of Yamanaka, similar approaches were used to manipulate cells in vitro and then transplant them in vivo for treating diseases. For example, Filareto et al. derived corrected dystrophic iPSCs from mice tail-tip fibroblasts missing dystrophin and utrophin, proteins implicated in muscle homeostasis. After generating myogenic progenitor cells from differentiation of corrected dystrophic iPSCs, they transplanted the cells into the muscles of mice with muscular dystrophy [75]. Immunofluorescence of muscle sections showed cell engraftment and expression of utrophin. This cell-based therapy led to some improvements in muscle function, but more efficient vectors are required to deliver reprogramming and differentiation factors and improve regeneration outcomes.

Viral-mediated transduction has been widely used in cell reprogramming, but the potential cytotoxicity an immunogenicity of viruses [76] has led to the development of non-viral vectors (nanocarriers) to deliver cargos inside cells [77]. Dexamethasone (Dex), a water-insoluble glucocorticosteroid, has been used as a supplement in osteogenic medium to induce the differentiation of mesenchymal stem cells (MSCs) into the osteoblastic lineage. After cell internalization, Dex binds to glucocorticoid receptors on the nuclei, resulting in their activation [78] and consequent expression of Runx2 gene [79] encoding a protein that is essential for osteoblastic differentiation. Dendrimers [80] and polymeric micelles [81] were used to entrap this hydrophobic molecule and allow its sustained release intracellularly in rMSCs. However, for in vivo delivery to specific cells and minimizing side effects in other tissues, targeting strategies are necessary. Santos et al. functionalized dendrimers with peptides, identified by phage display and with high affinity for MSCs, to deliver pDNA to those cells in vitro [82]. Using pDNA encoding enhanced green fluorescent protein (eGFP) and firefly luciferase (FLuc), successful transfection of cells was observed with low levels of cytotoxicity. Although transfection efficiency was superior in cells treated with peptide-functionalized dendrimers, cell selectivity was not demonstrated. A similar targeting strategy was developed using liposomes and phage-derived peptides targeting rMSCs to deliver sleeping beauty transposon plasmid [83]. To promote nuclear translocation, nuclear localization signal peptides were also incorporated in the nanocarrier system. The identified peptide was shown to be selective for rMSCs and improve transfection efficiency. The osteogenic differentiation of transfected MSCs was not affected, suggesting that gene delivery can be used to induce sustained gene expression in adult stem cells and enhance its therapeutic potential.

#### Promoting Tissue Regeneration In Vivo

In cell-based therapies, improving the properties of the niche where cells will be transplanted is essential to ensure cell engraftment. For example, inducing angiogenesis locally can promote skin wound healing and myocardial regeneration. Different approaches have been exploited to induce angiogenesis, including delivery of angiogenic factors (e.g., vascular endothelial GF—VEGF), transplantation of endothelial cells (ECs) or gene-based therapies, but it has been challenging to achieve a stable vasculature. Hubbell and co-workers designed a peptide-based vector (binding to DNA, nucleus and fibrin) to deliver pDNA encoding the stabilized variant of hypoxia-inducible factor, a transcription factor involved in the regulation of various pro-angiogenic factors [84]. The ability of the peptide-pDNA complexes embedded in a fibrin matrix to promote wound healing was tested in a mouse model of full-thickness dermal wound. Histological analysis showed increased number of ECs in wounds treated with peptide-pDNA system and VEGF-A_165_ (positive control), when compared with wounds treated with fibrin alone (negative control), indicating an enhanced angiogenic response. More mature vessels were observed in the wounds treated with peptide-pDNA system, as compared with controls, since smooth muscle cells were detected around the vessels. These results suggests the application of gene delivery as a strategy to achieve more controlled (physiological) angiogenesis and obtain more mature vascular structures, but the efficiency of this gene therapy still requires to be confirmed in models of impaired wound healing (diabetic mice).

Reconstruction of large bone defects continues to be a major clinical challenge, even when using autologous bone grafts. To promote osteointegration of bone implants, a liposome carrier was used to deliver cDNA for bone morphogenetic protein 2 (BMP-2) into peri-implant bone defects [85]. Immunocytochemistry analysis showed the presence of BMP-2 in cells migrating into the defects, demonstrating the successful transfection of cells in vivo. Bone regeneration was significantly enhanced in the defects treated with liposomes carrying BMP-2 gene, but not all the regions of the defect showed complete bone healing. It was postulated that this might have resulted from the insufficient number of cells mobilized to certain regions of the defect and not due to low transfection efficiency. This suggests the need for a homing strategy to recruit stem cells into the defect site using carriers functionalized with stem cell-binding peptides derived by phage display [86].

**Fig. 3:**
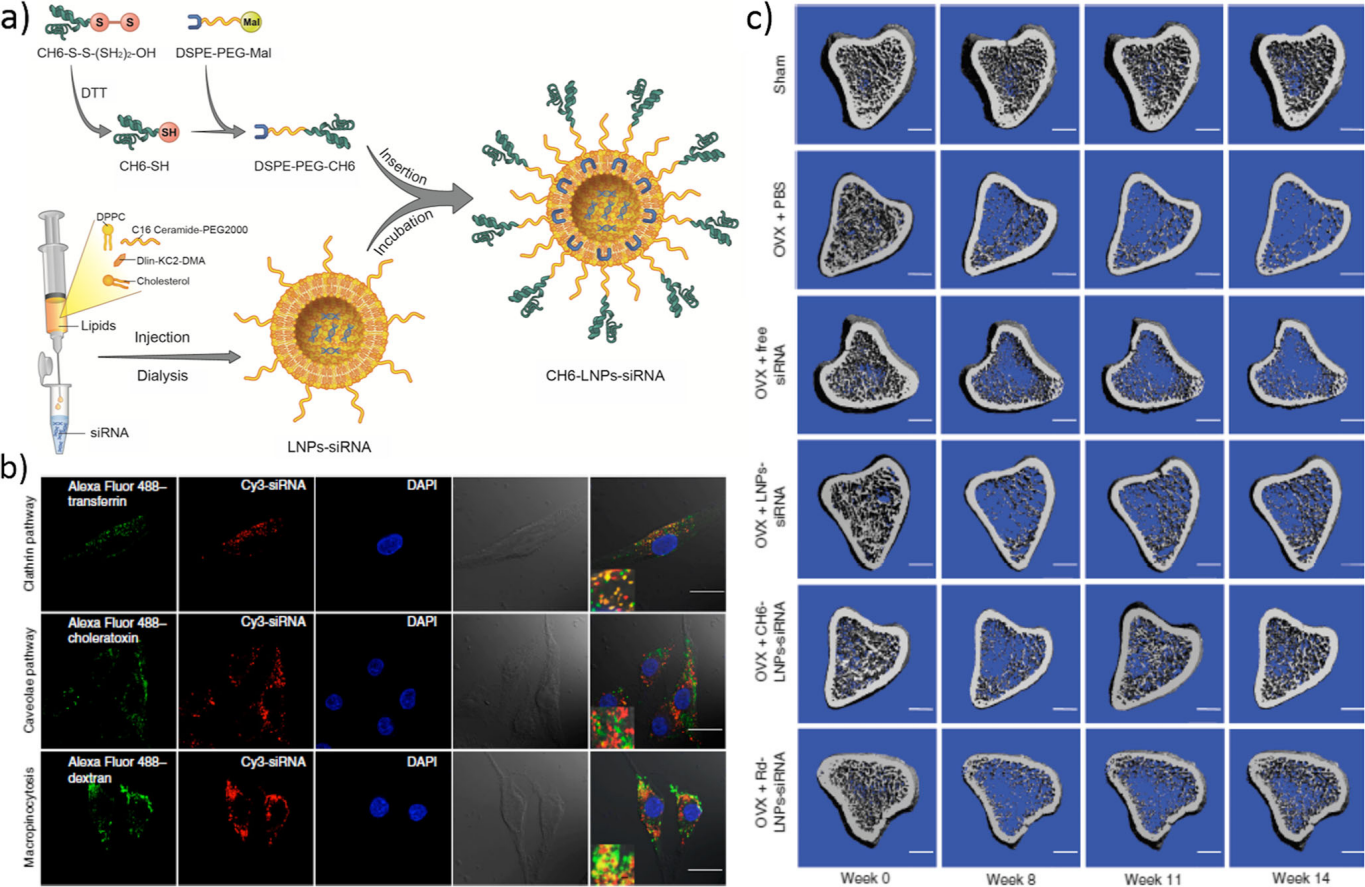
Stimulation of bone regeneration through targeted carrier-mediated delivery of genes silencing inhibitors of osteogenesis: **a** Schematic illustrating the preparation of lipid nanoparticles (LNPs), small interference RNAs (siRNA) loading and insertion of CH6 aptamer specific to rat osteoblasts; **b** Confocal fluorescence (first, second and third panels), bright-field (fourth panel) and merged (fifth panel) microscopy images of primary rat osteoblasts showing internalization of CH6-siRNA-LNPs; siRNA is stained in *red*, endocytic markers (transferrin, choleratoxin, dextran) in *green* and nuclei in *blue*; (*scale bar* =25 μm); **c** In vivo microcomputed tomography images of the proximal tibia of osteopenic ovariectomized (OVX) rats showing the 3D microstructure of bone before and after administration of different siRNA formulations (scale bar =1 mm). (Reprinted by permission from Macmillan Publishers Ltd.: Liang C, et al. Nat Med. 2015;21(3):288–94) [89••]

Metabolic bone disorders, such as osteoporosis, are characterized by abnormal calcium metabolism and/or bone cell physiology, leading to bone loss and skeletal failure. Osteoporosis drugs typically inhibit bone resorption, preventing bone loss, but they also slowdown the process of bone formation (osteogenesis). Using cationic liposomes functionalized with a peptide known to bind to calcified tissues of lower crystallinity, Zhang and co-workers were able to deliver small interference RNAs (siRNAs) specifically to bone-forming surfaces (lower crystallinity) in vivo [87]. By silencing the Plekho1 gene, that encodes a protein known to be a negative regulator of bone formation [88], they were able to stimulate bone formation. This delivery strategy was further upgraded by including a DNA aptamer specific to rat osteoblasts (CH6) on the surface of lipid nanoparticles (LNPs, Fig, 3a) [89••]. The aptamer-functionalized LPNs loaded with Plekho1 siRNA were shown to be internalized by osteoblasts in vitro (Fig. 3b). In vivo studies using osteopenic rats (low bone mineral density) showed enhanced bone formation with improved microarchitecture when rats were treated with CH6-siRNA-LNPs (Fig. 3c) as compared with other groups. By selectively stimulating bone formation without promoting bone resorption, this therapy holds promise to treat osteoporosis. However, further experiments are required to investigate possible off-target effects of this delivery strategy and determine the duration of the silencing effect.

### Imaging Techniques for Regenerative Medicine

**Fig. 4:**
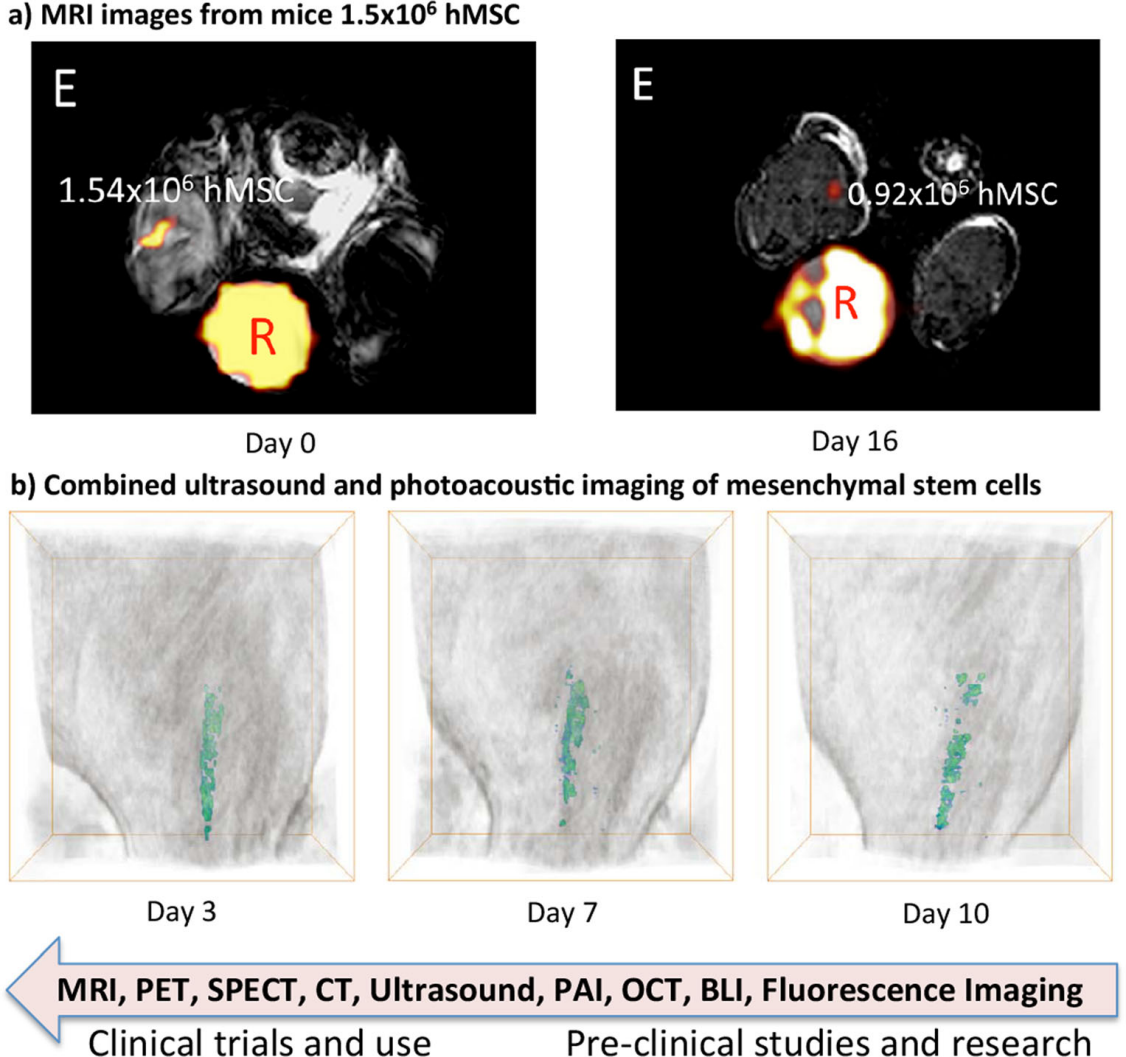
Examples of imaging modalities for RM and stem cell therapy: **a** Representative MR images for mice receiving hMSc at Day 0 and Day 16. F-MRI quantification correlates very well with the number of implanted cells at Day 0 and shows detectable hMSc at Day 16 [95]. **b** 3D longitudinal in vivo images by combined ultrasound and photoacoustic imaging (PAI) method of Au nanotracer-labeled mesenchymal stem cells [96]. Examples of BLI images can be seen in [97]. The *arrow* shows the closeness of these imaging modalities to clinical stem cell treatment

Imaging technologies offer a number of new opportunities in RM, for example, in the assessment of the tissue composition of organs, in transplanted cells monitoring or in cell therapy evaluation. The imaging modalities currently being used in stem cell therapies and research can be classified according to whether the targets are labeled with markers and whether the monitoring can be achieved in vivo [86, 90–93, 94•]. In this section, we will present an overview of the state-of-the-art imaging technologies in stem cell research and RM with the emphasis placed on in vivo imaging technologies that are currently being used or are likely to be adopted in clinical cell therapies (Fig. 4).

#### In Vivo Imaging Technologies in Clinical Use

Several matured clinical imaging technologies have good potential in clinical stem cell therapies. (i) Magnetic resonance imaging (MRI) is a very promising clinical imaging method [98–100] (Fig. 4a). The majority MRI signals are generated by the nuclear polarization on hydrogen atoms introduced by the strong magnetic field. This effectively shows the water distributions. MRI is very safe to use and is considered as the most robust imaging technology in clinical settings. MRI can be applied with contrast agent injection or without any exogenous labels, and has no depth limitation. (ii) Positron emission tomography (PET) injects a positron-emitting radioactive isotope incorporated in a metabolically active molecule such as fluorodeoxyglucose (FDG) [101–103]. In the circulating blood, FDG decays by emitting a positron, which meets an electron and produces two photons moving in opposite directions. PET has a very high sensitivity and has no limits in imaging depth and therefore can be used for tracing cell expressing reporter proteins, but requires ionizing radiation with associated biohazardous labels. In addition, rapid image acquisition (usually within a day) is needed for PET due to the short lifetime of the radioligands. (iii) Single photon emission tomography (SPECT) is a tomography technology and uses gamma rays for image acquisitions [104, 105]. By collecting 2D images using a gamma camera, 3D images can be reconstructed based on multiple 2D scans. SPECT has very high sensitivity and the tissue penetration depth is not limited but the spatial resolution is relatively low. Similar to PET, SPECT needs ionizing radiations, which may impose radiation risks. The main limitation of SPECT is that the short lifetime of the SPECT agent makes it only suitable for short-term cell tracing. (iv) Computed tomography (CT) sent X-ray beams (or proton beam and synchrotron X-rays) through an object and the beams are subsequently attenuated differently by various structures in the object according to their densities [106, 107]. The object 2-D slice can then be reconstructed through computer processing. Similar to MRI, CT has no limitations on imaging depth. However, the use of ionizing radiation such as X-rays to a certain extent limits the application in RM. (v) Ultrasound imaging uses acoustic waves from 2 to 13 MHz to acquire real-time images [96, 108, 109] (Fig. 4c). Despite a widely used clinical imaging technology offering rapid imaging solutions in clinical environment, the application of ultrasound imaging in RM is limited, due to its low image resolution and depth.

#### In Vivo Imaging Technologies for Pre-clinical Research

In addition to the clinical imaging technologies above, there are several other important research and pre-clinical imaging modalities for RM. (i) Photoacoustic imaging (PAI) is realized by using laser excited ultrasound waves to generate 3D images of soft tissues [110–112]. As a hybrid approach, PAI has the high contrast and good specificity offered by optical methods and the high spatial resolution and deeper penetration depth provided by the ultrasound modality. In RM PAI, gold nanoparticles used to label stem cells, can be tuned to have strong plasmon resonance at the excitation wavelengths to enhance the photoacoustic signal. (ii) Optical coherence tomography (OCT) uses low coherence light to acquire multiple 2D images through interferometry to reconstruct 3D images [113–115]. Although in most applications OCT is used as a label-free imaging technology, for stem cell research, the application of OCT relies on exogenous contrast agents such as magnetic and iron oxide particles, proteins, dyes, and nanomaterials to enhance the detection sensitivity to molecular level. (iii) Bioluminescence imaging (BLI) utilizes native light emissions from several bioluminescent organisms, e.g., the substrate D-luciferin by the enzyme Fluc from the North American Firefly [116–118] (Fig. 4d). BLI is a low-cost approach offering both high signal-to-noise ration and sensitivity, and has been widely used in small animal studies. The applications of BLI include tracking hematopoietic stem cell engraftment and assessing stem cell types. However, the strong scattering and absorption of the tissue limits the penetration depth of BLI. (iv) Fluorescence imaging uses an external light source to excite fluorescence emission from a range of fluorescent labels, such as green or red fluorescent proteins (GFP or RFP) and quantum dots (QD) [119–121]. The fluorescence image can be acquired by collecting the fluorescence emissions from the fluorophores. Similar to BLI, the penetration depth of fluorescence imaging is limited as a result of the strong absorption and scattering of the fluorescent light by the mammalian tissues.

#### Challenges and Opportunities in Stem Cell Imaging

For the in vivo imaging technologies reviewed above, to some extent, they are limited by a number of factors, such as the radiation hazard, the imaging depth limited by the tissue absorption and scattering, and reduced cell tracking time. These limitations result in low sensitivity and specificity, and limited ability to monitor cell changes and therapy progresses over time. Multimodality imaging is one promising direction to address such challenges, which combines multiple imaging methods to achieve an improved imaging performance [122]. For example, high-sensitivity and low-resolution methods such as PET can be used together with MRI to improve the image resolutions. For imaging modalities with a low penetration depth such as fluorescence imaging and PLI, one possible solution is to use implantable endoscopic imaging probes that can send down excitation light sources and at the same time to collect fluorescence emission from the fluorophores [123–125]. Alternatively, implantable imaging sensors can be embedded in the tissue or organ to collect real-time images and communicate with an external device through wireless signals [126, 127]. It is foreseeable that in vivo imaging will play an important role in future RM and may be performed routinely in clinical stem cell therapies throughout the course of treatment.

### Computational and Mathematical Modeling in Regenerative Medicine

The ability of mathematical and computational models to guide experimental discovery is increasingly appreciated in the field of RM, where the term “in silico” is more and more used when discussing the future of this promising field [128–130]. This section discusses recent opportunities that could make computational and mathematical modeling an important pillar of future RM research.

It is worthwhile to first emphasize why computational and mathematical modeling is important in the field of RM. Regenerating organs requires the ability to recreate a suitable physical and biochemical environment for the cells to grow. Elements of such environment include the mechanical and geometric properties of the growth substrate, the transport properties determining the rate at which nutrients and oxygen are supplied, and waste removed, and the level of mechanical load and fluid shear [130–132]. Without the framework provided by mathematical models, it would neither be possible to quantify and predict these properties nor to establish a link between macroscopic variables—which can be observed and controlled in an experiment—and microscopic variables that affect cell fate directly.

**Fig. 5:**
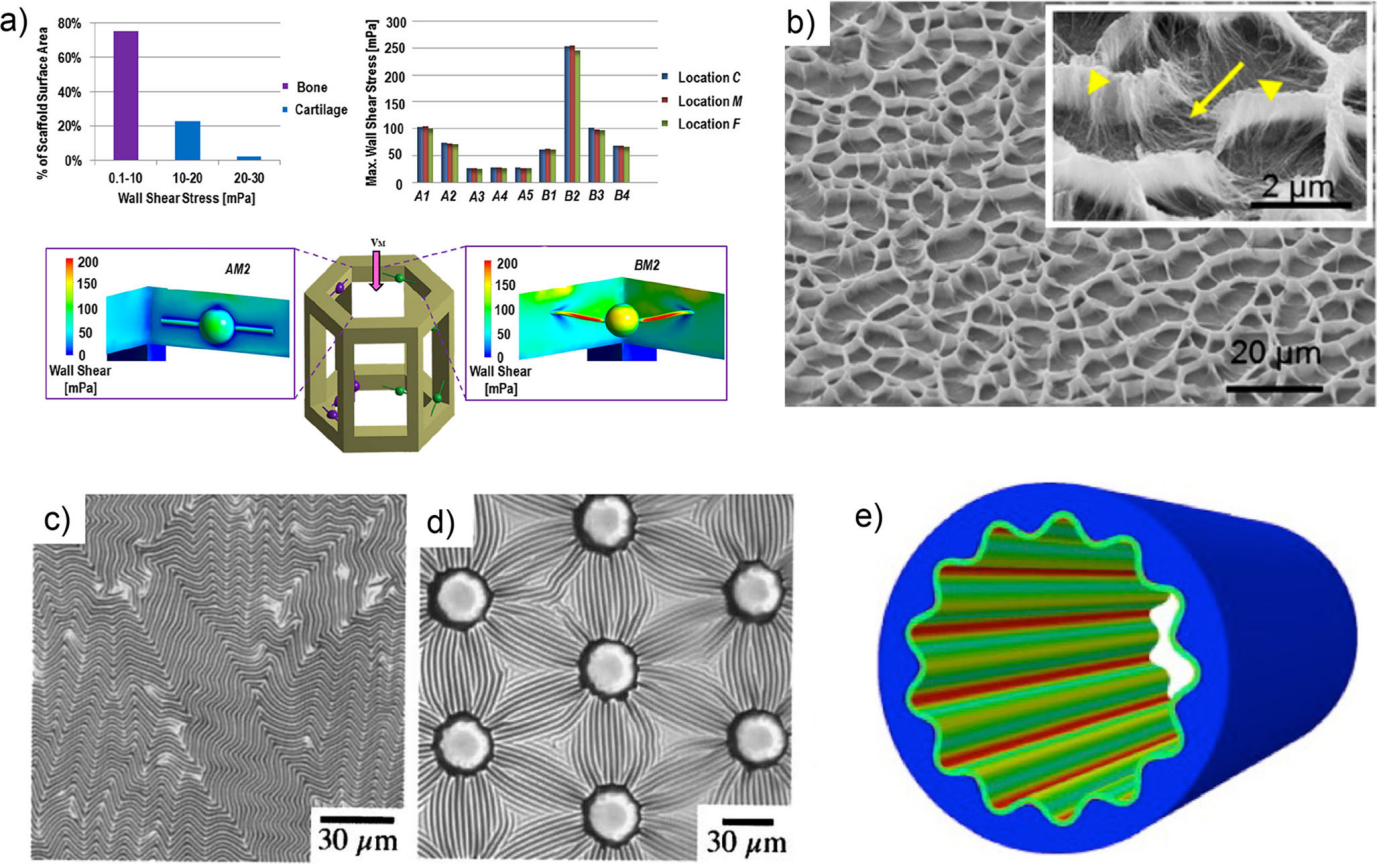
**a** Modeling based on computational fluid dynamics simulation of a scaffold for bone regeneration. (From: Zhao F, et al. Biomechanics and Modeling in Mechanobiology. 2015;14(2):231–43, with permission of Springer) [133]; **b** Scaffold for mesenchymal stem cells formed by elasto-capillary coalescence of carbon nanotube fibers (*arrow heads*: collapsed and laid CNTs, *arrows*: CNTs that were shrunk to form the razor-sharp peaks) (Reprinted from: Bitirim VC, et al. Materials Science and Engineering: C. 2013;33(5):3054–60, with permission from Elsevier) [134]. **c, d** Surface patterning through thin soft layer wrinkling. (Reproduced from: 137. Genzer J, Groenewold J. Soft Matter 2006;2(4):310–23, with permission of The Royal Society of Chemistry) [135]. **e** Finite element simulation of a buckled cylindrical shell. (Reprinted from: Li B, et al. Journal of the Mechanics and Physics of Solids. 2011;59(4):758–74, with permission from Elsevier) [136]

An example of this link is offered by the development of bioengineering scaffolds. While the ambient concentration of oxygen and nutrient can be easily controlled in experiment, and the average flow rate permeating through the scaffold adjusted, the local perfusion and shear stress level experienced by each cell will depend on the microscopic geometry of the scaffold, as well as the nutrient utilization by surrounding cells [130]. This coupled transport-mechanics phenomenon is impossible to disentangle by simple mechanicistic arguments. However, it lends itself perfectly to computer implementations (Fig. 5a) [133]. Validated simulations can offer insights, and be used to establish engineering correlations, by for example, providing the local transport environment experienced by each single cell as a function of flow rate and ambient concentration.

Today, purely mechanical phenomena related to bioengineering scaffolds can be simulated accurately. The wide availability of software to convert CT scans into a computer mesh (e.g., mimics), and to solve the relevant fluid and solid mechanics equations (e.g., fluent, abacus), renders the implementation of a simulation for optimizing the scaffold’s microstructure within reach of any modern bioengineering lab. The challenge that will probably occupy computational scientists in the future is the development—and validation against experiments—of suitable models for cell growth, mobility and interaction [132, 137, 138].

Two opportunities have emerged recently which could spur innovation in the development of substrates for cell growth and models of cell behavior. One is the growth of the discipline of soft matter physics and engineering. Such discipline, which is uniquely fitted to describe the fragile and water-filled structures where cells thrive, seeks to establish the physical principles governing the behavior of materials formed by soft macromolecular and colloidal elements, including the coupling of deformation mechanics, chemical reactions, fluid flow, physicochemical effects such as swelling, phase change, etc. Thanks to development in this exciting discipline, our ability to rationally design materials with micro/nanostructure that favours the growth of cells and the ability to characterize the behavior of cells has increased dramatically [139].

A recent example where soft matter has produced important results is the development of surface micro-patterning technique to promote cell colonization. Lithographic techniques can produce geometric features with exquisite control over microstructural geometry. However, deploying these techniques to produce cheaply and on large-scales surface micropatterns on practical scaffolds is a major challenge (e.g., lithographic techniques are not suitable to pattern the curved surfaces present in the interior of three-dimensional scaffolds). Fluidic phenomena and capillarity could offer an alternative route to micro-patterning. For example, the capillary forces produced by evaporating liquid films can produce remarkably regular patterns in fiber mats (Fig. 5b) [134]. The geometry of these patterns can be accurately predicted based on the theory of elasto-capillary coalescence, which has been the subject of increasing interest by the soft matter community recently [140]. Patterning through wrinkling (Fig. 5c–e) is another approach that heavily relies on recent applications of non-linear mechanics theories to soft matter systems [135]. Such theories enable to predict the wavelength of the wrinkles with extraordinary precision, enabling for instance to design the spacing and stiffness of protrusion where cells can anchor themselves. In addition to provide insights and predictions on how to make new materials, mathematical and computational modeling of soft matter provide ways to characterize the deformation of cells, enabling for instance to quantify the ability of cells to produce blebs that can aid their motility [141•, 142, 143] or cell-substrate adhesion phenomena [144].

A second opportunity is the realization of the importance of algorithmic mining of data as a predictive engineering tool in various areas of bioengineering [145, 146]. Data analysis is not a new discipline. What is new is the availability of inexpensive sensors (pressure, concentration, optical signals, etc.) that can be connected to equally inexpensive devices (e.g. smartphones, microfluidics kits, and Arduino boards) to interrogate in real-time the behavior of cultured cells, tissues, and organs. For example, one could conceive devices in which algorithms analyze multi-point data of cell motility from different positions in a cell monolayer and relate these to perfusion data extracted from simulations. The advent of “lab-on-a-chip” technologies [147, 148] makes the real-time interrogation of the data from micro-devices containing cells, and the feedback of this information to control the device’s operating parameters two important elements that require modeling. We believe that the convergence of cheap and robust sensing and imaging technologies, big data techniques, and physics-based computations could bring a new level of understanding of the complexity inherent in RM constructs, particularly in situations in which purely deterministic approaches have failed to provide sufficiently accurate predictive capabilities.

## Conclusion

Novel bioengineering technologies are redefining how we view and tackle key challenges in RM. We have provided a general overview of five dynamic and increasingly evolving areas that are shaping the next generation of RM therapies aiming to provide more selective cell-material interactions, higher precision, faster monitoring, more selective delivery, and more accurate predictions. Throughout these different areas, a general theme is the continuous push to improve precision, sensitivity, and selectivity; which are bringing us closer to the development of personalized RM.
